# Efficacy and safety of canakinumab in systemic juvenile idiopathic arthritis, the first Chinese experience

**DOI:** 10.1186/s12969-024-00974-4

**Published:** 2024-03-19

**Authors:** Lingzhi Qiu, Le Ma, Yifan Xie, Jing Jin, Yuting Pan, Shumin Li, Zhidan Fan, Haiguo Yu

**Affiliations:** Department of Rheumatology and Immunology, Children’s Hospital of Nanjing Medical University, No. 72 Guangzhou Road, Nanjing, Jiangsu Province 210008 China

**Keywords:** Canakinumab, Interleukin-1, Systemic juvenile idiopathic arthritis

## Abstract

**Background:**

Systemic juvenile idiopathic arthritis (sJIA) is a severe form of juvenile arthritis that is characterized by chronic joint inflammation and systemic symptoms such as fever, rash, and organ involvement. Anti-IL-6 receptor monoclonal antibody tocilizumab is an effective treatment. However, some patients still experience persisting or recurrent symptoms and the real-world effectiveness of canakinumab in Chinese patients with sJIA has never been reported. Therefore, this study aimed to assess the efficacy and safety of canakinumab in Chinese patients with sJIA using real-world data.

**Methods:**

We conducted a retrospective study on children with active sJIA. Clinical features, laboratory data, corticosteroid dosage, and adverse events (AEs) were collected at baseline and at 4, 8, 12, and 24 weeks after initiating canakinumab treatment.

**Results:**

Seven female and four male patients were included in the study. All patients had previously been treated with tocilizumab and were administered canakinumab for 12.4 ± 3.4 months. Notably, significant improvements were observed in both clinical signs and symptoms as well as laboratory indicators. Four children under corticosteroid treatment were able to successfully discontinue their corticosteroid therapy: one at week 4, two at week 12, and one at week 24. Notably, there was a significant reduction in the number of tender and swollen joints (*P* = 0.0059) as well as the systemic juvenile arthritis disease activity score (*P* < 0.0001). The most common AE was infection, but no patients experienced serious AEs. No cases of macrophage activation syndrome or death were reported during the follow-up period.

**Conclusions:**

Canakinumab was found to be potentially efficacious and safe in Chinese patients with sJIA. No new AEs were observed with canakinumab treatment.

## Background

Systemic juvenile idiopathic arthritis (sJIA), which accounts for 10–20% of all cases of juvenile idiopathic arthritis (JIA), is the most severe subtype of JIA. Chronic arthritis, systemic inflammation, fever, “salmon pink” rashes, lymphadenopathy, hepatosplenomegaly, and polyserositis are the primary characteristics of JIA. Approximately 10% of patients with sJIA develop a potentially fatal complication called macrophage activation syndrome (MAS) [1].

The treatment goals for sJIA are achieving and maintaining complete clinical remission, preventing complications, and improving long-term prognosis. The development of inflammatory cytokine-targeted therapies has remarkably changed the treatment of sJIA [2]. Increasing evidence suggests that interleukin (IL)-1 and IL-6 play crucial roles in the pathogenesis of sJIA [3–5]. There appears to be a "window of opportunity" to prevent the development of chronic arthritis by utilizing IL-1 or IL-6 blockade as a first-line treatment [6–10]. Canakinumab, a selective, fully human anti-IL1β monoclonal antibody, has been found to be safe and effective in treating patients with sJIA [1, 11–15]. Both the U.S. Food and Drug Administration (FDA) and European Medicines Agency (EMA) have approved canakinumab for the treatment of sJIA with active systemic features in patients aged at least 2 years and weighing at least 7.5 kg [16, 17]. To the best of our knowledge, this is the first study to investigate the efficacy and safety of canakinumab in Chinese patients with sJIA and to evaluate its therapeutic potential. All study participants had experienced tocilizumab treatment failure before enrollment.

## Methods

This retrospective observational study was conducted at a single center and involved 13 patients diagnosed with sJIA who underwent treatment with canakinumab between November 2021 and May 2023. These patients had a minimum follow-up duration of 24 weeks while receiving canakinumab treatment. However, two patients were excluded from the analysis due to loss of follow-up after only one treatment; hence, no follow-up data were available. All patients were less than 16 years old, with a confirmed diagnosis according to the International League of Associations for Rheumatology (ILAR) classification criteria for sJIA [18]. Whole exon gene sequencing was performed to exclude other autoinflammatory diseases.

The study was approved by the Ethics Committee of Children’s Hospital of Nanjing Medical University (approval number: 202211257-1). The legal guardians of the children signed written consents for the medication; however, the need for informed consent for study participation was granted exemption due to the retrospective nature of the study. We reviewed patient’s medical records, collected relevant data, and selected clinical visit records from electronic outpatient medical records as well as medical record files during hospitalization (discharge abstract, progress notes, and medical reports). This retrospective analysis covered demographic, clinical, laboratory, and treatment-related data, including age, age at onset, sex, duration from onset to receiving canakinumab treatment, duration of exposure to canakinumab, dosage used, previous and concomitant treatments, previous MAS, number of tender and swollen joints, systemic Juvenile Arthritis Disease Activity Score 27 (sJADAS 27) and adverse events (AEs). The analysis of laboratory parameters for disease activity was carried out in the hospital laboratory. The current diagnostic criteria for MAS still refer to the preliminary diagnostic guidelines for sJIA with MAS proposed by Ravelli [19]. The sJADAS 27 was made up of five aspects: physician global assessment of disease activity, parent or patient global assessment of disease activity, the count of active joints in 27 joints, normalized ESR, and clinical manifestations. The sum of the five scores composed and scored the sJADAS 27 score [20]. Safety of canakinumab therapy was assessed using the AE, i.e., any untoward medical events that occurred immediately after or during drug administration.

The study endpoints included clinical and laboratory manifestations at week 4, 8, 12, and 24 of the follow-up visits. The selected clinical visits were documented closer to these time points.Further endpoints included a statistically significant decrease in the number of tender and swollen joints, corticosteroid dosage, and sJADAS 27.

### Statistical analysis

Characteristics were summarized using descriptive statistics, including sample sizes, means, and standard deviations. Statistical computations were performed using GraphPad software, and graphical representations were generated. Statistical analysis was performed using the one-way analysis of variance with post hoc Bonferroni’s test for intra-group comparisions to baseline values. *P*-values < 0.05 were considered significant.

## Results

A total of 11 patients treated with canakinumab for active sJIA had follow-up data for 24 weeks of treatment. Table 1 summarizes the patients’ clinical and laboratory characteristics at the initiation of canakinumab treatment (baseline). The mean age at disease onset was 4.8 ± 2.6 years, and 63.6% (*n* = 7) were female. The average duration from disease onset to receiving canakinumab treatment was 49.6 ± 42.5 months. In more than half of the patients (63.6%), the duration of sJIA before the commencement of canakinumab treatment exceeded 2 years. At baseline, eight (72.7%) patients presented with systemic manifestations such as fever, rash, lymphadenopathy, and myalgia. Arthritis (*n* = 10; 90.9%), fever (*n* = 7; 63.6%), rash (*n* = 3; 27.3%), and myalgia (*n* = 3; 27.3%) were the most common symptoms. Of the 10 patients with active arthritis, five (50%) had active inflammation in one to four joints. Additionally, three patients experienced episodes of MAS before starting the canakinumab treatment. Whole exon gene sequencing was performed in seven cases to exclude other autoinflammatory diseases. Lack of efficacy was the primary reason for discontinuing previous treatment regimens, leading to the switch to canakinumab in all patients.

**Table 1:** Clinical and laboratory characteristics at baseline

Variables	Case1	Case2	Case3	Case4	Case5	Case6	Case7	Case8	Case9	Case10	Case11
Sex	F	F	M	M	F	M	F	F	F	M	F
Age at disease onset (years)	3.67	9.5	3.33	3.58	1.92	4.42	3.92	5.42	6	1.58	9.1
Age at diagnosis (years)	4.25	9.5	3.42	6.58	2	4.42	4.1	5.42	6	2	9.25
Time from onset to receiving canakinumab (months)	9	9	15	100	48	45	42	23	37	148	69
Number of joints with active inflammation	10	2	6	1	3	3	0	2	12	21	5
Fever	+	_	+	+	+	+	+	+	_	_	_
Rash	_	_	_	_	_	+	+	+	_	_	_
Lymphadenopathy	+	_	_	+	_	+	_	_	_	_	_
Myalgia	+	+	_	_	_	+	_	_	_	_	_
Previous MAS	_	_	_	_	+	_	+	+	_	_	_
Ferritin > 330 ng/mL	+	_	+	+	+	+	_	_	_	+	_
WBC > 15,000/mm^3^	+	+	+	+	+	+	+	+	_	_	+
ESR > 20 mm/h	+	_	+	+	+	_	+	+	+	+	+
CRP > 10 mg/L	+	_	+	+	+	+	+	+	+	+	+
Increased transaminases	_	_	_	_	_	+	_	_	_	_	_

*F* Female, *M* Male, *MAS* Macrophage activation syndrome, *WBC* White blood cell, *ESR* Erythrocyte sedimentation rate, *CRP* C-reactive protein

Table 2 shows previous and concomitant treatments at baseline. All 11 patients had previously received non-steroidal anti-inflammatory drugs (NSAIDs), corticosteroids, conventional disease-modifying antirheumatic drugs (cDMARDs), and biological disease-modifying antirheumatic drugs (bDMARDs). Tocilizumab (*n* = 11, 100%), adalimumab (*n* = 5, 45.5%), and etanercept (*n* = 3, 27.3%) were the most common biological treatments preceding canakinumab initiation. At baseline, nine (81.8%) patients were treated with NSAIDs, four with corticosteroid, and nine with non-IL-1 inhibiting biologicals. Ten (90.9%) patients received at least one cDMARD, with three concurrently receiving two or more cDMARDs. Methotrexate (MTX) was the most commonly used cDMARDs at baseline (90.0% MTX and 20.0% thalidomide). Seven patients treated with tocilizumab and two patients with adalimumab switched to canakinumab at baseline. The patient in Case 11 was treated with janus kinase inhibitors (JAKi) at baseline and discontinued JAKi immediately after receiving canakinumab. NSAIDs, corticosteroids and cDMARDs continued to be used after baseline treatment.

**Table 2:** Previous and concomitant treatments at baseline

Treatment	Case 1	Case 2	Case 3	Case 4	Case 5	Case 6	Case 7	Case 8	Case 9	Case 10	Case 11
**Previous treatment**											
NSAIDs											
Diclofenac sodium	**-**	**-**	**-**	**-**	**-**	**-**	**-**	**-**	**-**	**-**	**+**
Naproxen	**+**	**+**	**+**	**+**	**+**	**+**	**+**	**+**	**+**	**+**	**+**
Celecoxib	**-**	**-**	**-**	**+**	**-**	**-**	**-**	**-**	**-**	**-**	**-**
Corticosteroid											
Corticosteroid	**+**	**+**	**+**	**+**	**+**	**+**	**+**	**+**	**+**	**+**	**+**
Corticosteroid pulse	**+**	**-**	**-**	**-**	**+**	**-**	**+**	**+**	**-**	**-**	**-**
cDMARDs											
Methotrexate	**+**	**+**	**+**	**+**	**+**	**+**	**+**	**+**	**+**	**+**	**+**
Cyclosporine A	**-**	**-**	**-**	**-**	**-**	**-**	**-**	**+**	**-**	**-**	**-**
Leflunomide	**+**	**-**	**-**	**+**	**+**	**+**	**+**	**+**	**-**	**-**	**+**
Thalidomide	**+**	**+**	**-**	**+**	**-**	**+**	**+**	**+**	**+**	**-**	**+**
Sulfasalazine	**-**	**-**	**-**	**+**	**-**	**-**	**-**	**-**	**-**	**-**	**-**
bDMARDs											
Tocilizumab	**+**	**+**	**+**	**+**	**+**	**+**	**+**	**+**	**+**	**+**	**+**
Adalimumab	**-**	**-**	**-**	**+**	**+**	**-**	**-**	**-**	**+**	**+**	**+**
Etanercept	**-**	**-**	**-**	**+**	**-**	**+**	**-**	**-**	**-**	**+**	**-**
JAKi	**-**	**-**	**-**	**-**	**-**	**-**	**+**	**-**	**-**	**-**	**+**
**Treatment at baseline**											
NSAIDs	**+**	**+**	**+**	**+**	**-**	**+**	**-**	**+**	**+**	**+**	**+**
Corticosteroid	**+**	**-**	**-**	**+**	**+**	**-**	**-**	**+**	**-**	**-**	**-**
cDMARDs											
Methotrexate	**+**	**+**	**+**	**-**	**+**	**+**	**-**	**+**	**+**	**+**	**+**
Leflunomide	**-**	**-**	**-**	**+**	**-**	**-**	**-**	**-**	**-**	**-**	**-**
Thalidomide	**-**	**-**	**-**	**-**	**-**	**-**	**-**	**-**	**+**	**-**	**+**
Sulfasalazine	**-**	**-**	**-**	**+**	**-**	**-**	**-**	**-**	**-**	**-**	**-**
bDMARDs											
Tocilizumab	**-**	**+**	**+**	**+**	**-**	**+**	**+**	**+**	**-**	**-**	**+**
Adalimumab	**-**	**-**	**-**	**-**	**+**	**-**	**-**	**-**	**+**	**-**	**-**
JAKi	**-**	**-**	**-**	**-**	**-**	**-**	**-**	**-**	**-**	**-**	**+**

*NSAIDs* Non-steroidal anti-inflammatory drugs, *cDMARDs* Conventional disease-modifying antirheumatic drugs, *bDMARDs* Biologic disease-modifying antirheumatic drugs, *JAKi* Janus kinase inhibitors

None of the patients discontinued canakinumab treatment during the follow-up period. The initial dosage of canakinumab ranged from 0.5 mg/kg/4 weeks to 4 mg/kg/4 weeks (maximum = 150 mg). One patient received an increased dose at week 12 due to persistent but improved laboratory and clinical inflammatory manifestations. The frequency of administration decreased in two patients, and the injection interval was extended to 45 days and 2 months, respectively. The overall duration of canakinumab treatment was 12.4 ± 3.4 months.

**Fig. 1:**
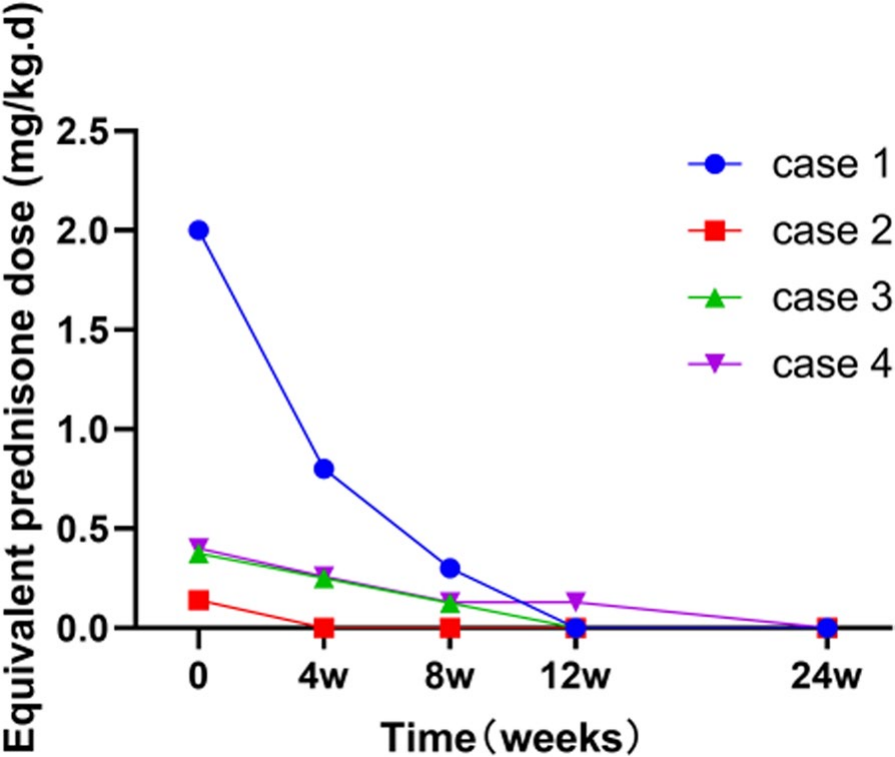
Corticosteroid tapering in the four patients at different follow-up visits

**Fig. 2:**
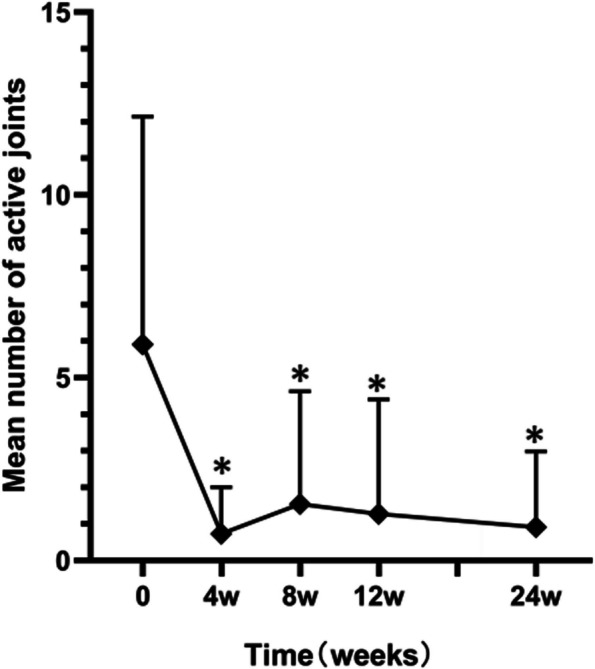
The trend in the mean number of active joints at different follow-up visits. *P < 0.05 vs. week 0

**Fig. 3:**
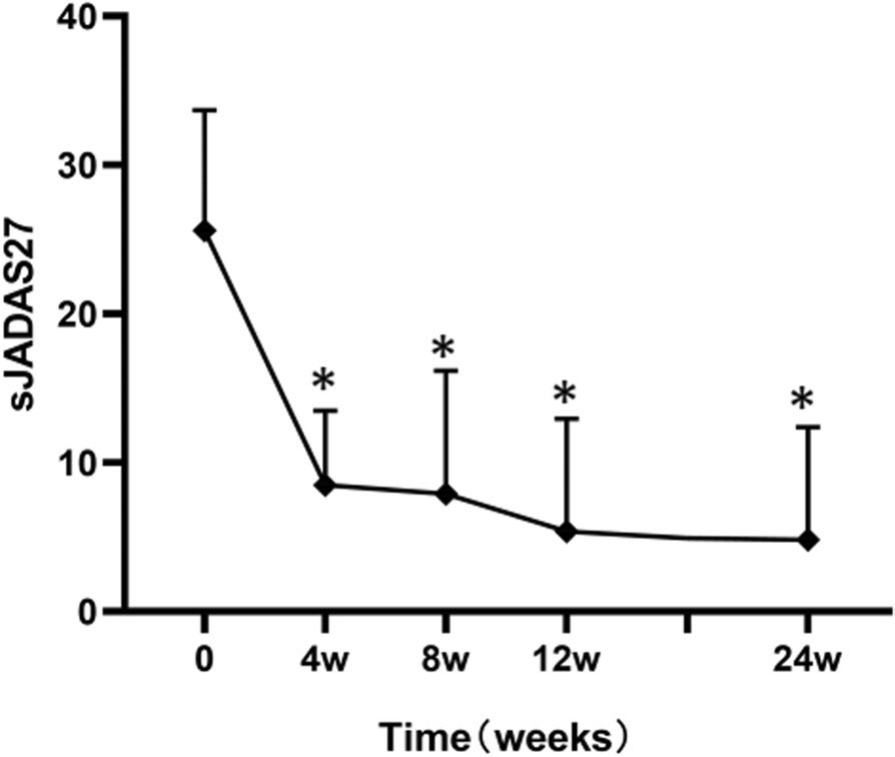
The trend of the disease activity score on the sJADAS 27 at different follow-up visits. *P < 0.05 vs. week 0

Clinical manifestations such as fever, arthritis, rash, and myalgia, along with increased levels of inflammatory indicators such as C-reactive protein (CRP), erythrocyte sedimentation rate (ESR), and ferritin were observed during different follow-up assessments (Table 3). Inflammatory indicators normalized within 12 weeks in nine of the 11 patients, and remained consistently low in the subsequent follow-up. At week 24, only one patient experienced fever due to pneumonia, whereas the others had no fever, rash, or myalgia. Four patients with corticosteroid use at baseline successfully reduced their corticosteroid dosage within 4 weeks. In addition, the proportion of corticosteroid-free patients at 12 and 24 weeks of canakinumab therapy was notably high, reaching 75% (3/4) and 100% (4/4), respectively. Corticosteroid tapering during canakinumab treatment is shown in Fig. 1. The number of tender and swollen joints was significantly reduced (*P* = 0.0059) during the study, declining from 5.9 at baseline to 0.9 at week 24 (Fig. 2). Additionally, as shown in Fig. 3, the mean sJADAS 27 decreased significantly from baseline to week 4, from a mean score of 25.6 to 8.5, respectively; moreover, this effect lasted until week 24 of the study.

**Table 3:** The specific clinical and laboratory manifestations at different follow-up visits in each patient

Clinical features	Case 1 (weeks)					Case 2 (weeks)					Case 3 (weeks)					Case 4 (weeks)					Case 5 (weeks)					Case 6 (weeks)				
	0	4	8	12	24	0	4	8	12	24	0	4	8	12	24	0	4	8	12	24	0	4	8	12	24	0	4	8	12	24
Fever	+	-	-	-	-	-	-	-	-	-	+	-	-	-	-	+	-	-	-	-	+	-	-	-	-	+	-	-	-	-
Rash	-	-	-	-	-	-	-	-	-	-	-	-	-	-	-	-	-	-	-	-	-	-	-	-	-	+	-	-	-	-
Lymphadenopathy	+	-	-	-	-	-	-	-	-	-	-	-	-	-	-	+	-	-	-	-	-	-	-	-	-	+	-	-	-	-
Myalgia	+	-	-	-	-	+	-	-	-	-	-	-	-	-	-	-	-	-	-	-	-	-	-	-	-	+	+	-	-	-
Arthritis	+	-	-	-	-	+	+	-	-	-	+	-	-	-	-	-	-	-	-	-	+	+	+	-	-	+	-	-	-	-
WBC > 15,000/mm^3^	+	-	-	-	-	+	-	-	-	-	+	-	-	-	-	+	-	-	-	-	+	-	-	-	+	+	-	-	-	-
CRP > 10 mg/L	+	-	-	-	-	-	-	-	-	-	+	-	-	-	-	+	-	-	-	-	+	+	-	-	+	+	-	-	-	-
ESR > 20 mm/h	+	-	-	-	-	-	-	-	-	-	+	-	-	-	-	+	-	-	-	-	+	-	-	-	-	-	-	-	-	-
Ferritin > 330 ng/mL	+	-	-	-	-	-	-	-	-	-	+	-	-	-	-	+	-	-	-	-	+	-	-	-	-	+	-	-	-	-

Clinical features	Case 7 (weeks)					Case 8 (weeks)					Case 9 (weeks)					Case 10 (weeks)					Case 11 (weeks)				
	0	4	8	12	24	0	4	8	12	24	0	4	8	12	24	0	4	8	12	24	0	4	8	12	24
Fever	+	-	-	-	-	+	-	-	-	-	-	-	+	-	+	-	-	-	-	-	-	-	-	-	-
Rash	+	-	-	-	-	+	-	-	-	-	-	-	-	-	-	-	-	-	-	-	-	-	-	-	-
Lymphadenopathy	-	-	-	-	-	-	-	-	-	-	-	-	-	-	-	-	-	-	-	-	-	-	-	-	-
Myalgia	-	-	-	-	-	-	-	-	-	-	-	-	-	-	-	-	-	-	-	-	-	-	-	-	-
Arthritis	-	-	-	-	-	+	-	-	-	-	+	-	+	+	+	+	+	+	+	+	+	+	+	+	-
WBC > 15,000/mm^3^	+	-	-	-	-	+	-	-	-	-	-	-	-	-	-	-	-	-	-	-	+	-	-	-	-
CRP > 10 mg/L	+	-	-	-	-	+	-	-	-	-	+	+	+	+	+	+	-	-	-	+	+	-	-	-	-
ESR > 20 mm/h	+	-	-	-	-	+	-	-	-	-	+	-	+	+	+	+	-	+	+	+	+	-	-	-	-
Ferritin > 330 ng/mL	-	-	-	-	-	-	-	-	-	-	-	-	-	-	-	+	-	-	-	-	-	-	-	-	-

*WBC* White blood cell, *CRP* C-reactive protein, *ESR* Erythrocyte sedimentation rate

The mean duration of exposure to canakinumab was 12.4 ± 3.4 months. A total of eight (72.7%) patients experienced 20 AEs. Specifically, eight patients experienced at least one AE during the study, resulting in an exposure-adjusted AE rate of 364 per 100 patient-years. The observed AEs included eight cases of viral upper respiratory tract infection and one case of pneumonia. Notably, none of the patients required discontinuation of canakinumab treatment. No instances of anaphylactic or injection site reactions were observed. Additionally, no deaths, MAS, or flares occurred during the 24 weeks treatment period with canakinumab.

## Discussion

To the best of our knowledge, this is the first study to present the efficacy and safety of canakinumab in Chinese patients with active sJIA. All study participants had previously experienced tocilizumab treatment failure with active sJIA, with symptoms such as fever, arthritis, elevated CRP, and ESR. No patient discontinued canakinumab treatment during follow-up. Corticosteroid-tapering effect was observed in all patients on corticosteroid therapy at baseline and remained low, with significant reductions in active arthritis as well as sJADAS 27.

sJIA accounts for 5–15% of all cases of chronic arthritis in children which presents a significant challenge in terms of diagnosis and treatment. Early biological therapy has been shown to improve the long-term prognosis of patients [8]. In our case, 72.7% (8/11) of the patients started using tocilizumab within the first year of treatment, indicating refractory cases. Extensive evidence suggests that pro-inflammatory cytokines IL-1, especially IL -1β, have been implicated as pivotal cytokines in sJIA [21]. Canakinumab has been found to be highly effective in the treatment of sJIA as it binds selectively to IL-1β, inactivates IL-1β signaling pathways, and neutralizes its downstream effects [1, 11–15]. It is the only anti-IL-1 agent licensed for the treatment of sJIA. Ruperto et al. [1] reported that after the first administration of canakinumab, 60% of the patients achieved an adapted ACR Pedi 50 at day 15. Double-blind, placebo-controlled trials also showed 64.4% of patients with canakinumab experiencing reduced disease activity within 6 months and 93.5% of patients within 2 years [14]. During the 24-week follow-up in the treatment of our patients, the remission of clinical symptoms and signs, as well as laboratory indicators, confirmed the beneficial effects of canakinumab. A continuous decline was observed in the mean sJADAS 27 and active joints at different follow-up time points. In addition, over 70% (8/11, with one case of infection) of patients at week 24 achieved complete absence of all signs and symptoms related to sJIA, such as fever, arthritis, and increased acute-phase reactant levels.

Avoiding or reducing corticosteroid use is one of the current treat-to-target treatment strategies for sJIA. Published clinical trials have demonstrated that 15.6%–55.6% of patients with sJIA treated with canakinumab were able to discontinue corticosteroid use [1, 12, 14, 15, 22, 23]. All participants in our study were previously on corticosteroids, and the median duration of corticosteroid therapy was 22 months (range: 3–95 months). Four patients were treated with corticosteroids at baseline, and the mean duration of exposure to corticosteroids was 21.8 ± 10.3 months. By week 4, all of them successfully tapered their corticosteroid dosage, and further corticosteroid dose reduction was achieved throughout the study. In addition, the number of corticosteroid-free patients increased from 1 (25%) at week 4 to 4 (100%) at week 24, suggesting a rapid and continuous corticosteroid-tapering effect of canakinumab. Considering the side effects of corticosteroid, the corticosteroid-sparing effect of canakinumab therapy holds crucial benefits for patients with sJIA.

Indeed a biphasic model of the disease has been proposed and early use of biologic agents has been advocated based on the hypothesis that this early approach could prevent the progression of chronic synovitis in sJIA. Studies have shown that giving anakinra early in the disease course appears to be more efficacious in achieving clinically inactive disease and less efficacious when given later [8, 24]. Data from the German Biologics register showed that patients treated with IL-1 blockades within 12 months of the disease commencement achieved clinical remission more frequently than patients who started treatment after the first 12 months [10]. Thses may support the “window of opportunity” hypothesis. However, in an open-label phase III study [15], canakinumab was efficacious and associated with substantial corticosteroid dose reduction in patients with sIJA whose median time from diagnosis to study entry was 5.9 (range: 0.4–17.3) years. In our cases, from the time of onset to receiving canakinumab, two cases were within 12 months and nine cases were over 12 months. However, after treatment with canakinumab, their clinical symptoms were obviously controlled and their inflammatory indicators were relieved. However, some patients failed in the treatment of IL-1 inhibition and achieved satisfactory results when switched to IL-6 inhibition, and vice versa. Whether this is due to true heterogeneity of the disease itself or different mechanisms at different stages remains to be established [25]. In this study, tocilizumab was replaced with canakinumab for the treatment of patients with JIA due to its lack of efficacy, which resulted in favorable outcomes for all patients. Several hypotheses may explain the lack of response to tocilizumab in patients with sJIA. First, as IL-6 is downstream of IL-1β, systemic inflammation and arthritis were observed in IL-1 but not IL-6 overexpressing transgenic mice in some studies [26, 27]. IL-6 is undetectable in IL-1β-deficient mice, whereas IL-1 is normally expressed in IL-6-deficient mice [28, 29]. Second, IL-1β concentration is not affected by tocilizumab [30], but canakinumab causes a rapid reduction of IL-1β-stimulated IL-6 secretion [31]. IL-6 levels decreased by day 3 and persisted for 24 and 48 weeks after canakinumab treatment [21].

MAS is a life-threatening complication of sJIA with a mortality rate of up to 20%. Some researchers reasonably expect that the frequency of MAS in sJIA can be potentially reduced through treatment with IL-1 blockades [11]. Kostik et al. [32] reported on eight patients with sJIA who developed MAS that was resolved by a short-term increase in canakinumab doses in all patients. In our study, three patients experienced four MAS events in the past, however, none developed MAS during canakinumab treatment.

The overall safety of canakinumab observed in this study was good and is consistent with that of previous studies [1, 12–15, 22, 33]. Sota et al. [34] reported an estimated AE rate of 8.4/100 patients/year. Discontinuation owing to AE is rare. In our cohort, 72.7% of patients experienced AEs during follow-up; however, none of the patients withdrew from the study due to AEs. The most commonly reported AE was infection, particularly upper respiratory infection. Notably, no novel safety findings were observed. Severe AEs, such as MAS and death, were not reported in the study.

However, this study has several limitations, including a small sample size, a short study period, and a retrospective design without standardized treatments. It was also limited by insufficient information on novel biomarkers such as, S100, IL-18, HO-1, MMP3, etc. Future studies should assess such biomarkers in order to provide clinical diagnosis, assess disease activity, and guide treatment.

## Conclusions

Our research adds to the evidence of the effectiveness and safety of canakinumab in patients with sJIA. This drug exhibits a remarkable ability to improve the clinical symptoms and laboratory indicators associated with sJIA, leading to a significant reduction in corticosteroid dosages while maintaining a positive safety profile.

## Data Availability

The datasets used and/or analyzed in the current study are available and can be obtained from the corresponding author on reasonable request.

## Abbreviations

sJIA: Systemic juvenile idiopathic arthritis
AEs: Adverse events
F: Female
M: Male
WBC: White blood cells
CRP: C-reactive protein
ESR: Erythrocyte sedimentation rate
MAS: Macrophage activation syndrome
sJADAS 27: Systemic Juvenile Arthritis Disease Activity Score 27
IL: Interleukin
FDA: U.S. Food and Drug Administration
EMA: European Medicines Agency
ILAR: International League of Associations for Rheumatology
NSAIDs: Non-Steroidal Anti-Inflammatory Drugs
cDMARDs: Conventional disease-modifying antirheumatic drugs
bDMARDs: Biologic disease-modifying antirheumatic drugs
JAKi: Janus kinase inhibitors

## References

[1] Ruperto N, Quartier P, Wulffraat N, Woo P, Ravelli A, Mouy R (2012). A phase II, multicenter, open-label study evaluating dosing and preliminary safety and efficacy of canakinumab in systemic juvenile idiopathic arthritis with active systemic features. Arthritis Rheum.

[2] Toplak N, Blazina Š, Avčin T (2018). The role of IL-1 inhibition in systemic juvenile idiopathic arthritis: current status and future perspectives. Drug Des Devel Ther.

[3] Bruck N, Schnabel A, Hedrich CM (2015). Current understanding of the pathophysiology of systemic juvenile idiopathic arthritis (sJIA) and target-directed therapeutic approaches. Clin Immunol.

[4] Gattorno M, Piccini A, Lasigliè D, Tassi S, Brisca G, Carta S (2008). The pattern of response to anti-interleukin-1 treatment distinguishes two subsets of patients with systemic-onset juvenile idiopathic arthritis. Arthritis Rheum.

[5] Pascual V, Allantaz F, Arce E, Punaro M, Banchereau J (2005). Role of interleukin-1 (IL-1) in the pathogenesis of systemic onset juvenile idiopathic arthritis and clinical response to IL-1 blockade. J Exp Med.

[6] Föll D, Wittkowski H, Hinze C (2020). Still’s disease as biphasic disorder: current knowledge on pathogenesis and novel treatment approaches. Z Rheumatol.

[7] Giancane G, Minoia F, Davì S, Bracciolini G, Consolaro A, Ravelli A (2016). IL-1 inhibition in systemic juvenile idiopathic arthritis. Front Pharmacol.

[8] Vastert SJ, de Jager W, Noordman BJ, Holzinger D, Kuis W, Prakken BJ (2014). Effectiveness of first-line treatment with recombinant interleukin-1 receptor antagonist in steroid-naive patients with new-onset systemic juvenile idiopathic arthritis: results of a prospective cohort study. Arthritis Rheumatol.

[9] Nigrovic PA (2014). Review: is there a window of opportunity for treatment of systemic juvenile idiopathic arthritis?. Arthritis Rheumatol.

[10] Horneff G, Schulz AC, Klotsche J, Hospach A, Minden K, Foeldvari I (2017). Experience with etanercept, tocilizumab and interleukin-1 inhibitors in systemic onset juvenile idiopathic arthritis patients from the BIKER registry. Arthritis Res Ther.

[11] Grom AA, Ilowite NT, Pascual V, Brunner HI, Martini A, Lovell D (2016). Rate and clinical presentation of macrophage activation syndrome in patients with systemic juvenile idiopathic arthritis treated with canakinumab. Arthritis Rheumatol.

[12] Ruperto N, Brunner HI, Quartier P, Constantin T, Wulffraat N, Horneff G (2012). Two randomized trials of canakinumab in systemic juvenile idiopathic arthritis. N Engl J Med.

[13] Feist E, Quartier P, Fautrel B, Schneider R, Sfriso P, Efthimiou P (2018). Efficacy and safety of canakinumab in patients with Still’s disease: exposure-response analysis of pooled systemic juvenile idiopathic arthritis data by age groups. Clin Exp Rheumatol.

[14] Ruperto N, Brunner HI, Quartier P, Constantin T, Wulffraat NM, Horneff G (2018). Canakinumab in patients with systemic juvenile idiopathic arthritis and active systemic features: results from the 5-year long-term extension of the phase III pivotal trials. Ann Rheum Dis.

[15] Nishimura K, Hara R, Umebayashi H, Takei S, Iwata N, Imagawa T (2021). Efficacy and safety of canakinumab in systemic juvenile idiopathic arthritis: 48-week results from an open-label phase III study in Japanese patients. Mod Rheumatol.

[16] Label and approval history for Ilaris. Washington (DC): FDA; 2014. https://www.accessdata.fda.gov/scripts/cder/daf/#apphist.

[17] European public assessment report (EPAR) for Ilaris. Brussels: European Medicines Agency; 2016. https://www.ema.europa.eu/en/medicines/human/EPAR/ilaris.

[18] Petty RE, Southwood TR, Manners P, Baum J, Glass DN, Goldenberg J (2004). International League of Associations for Rheumatology. International League of Associations for Rheumatology classification of juvenile idiopathic arthritis: second revision, Edmonton, 2001. J Rheumatol.

[19] Ravelli A, Magni-Manzoni S, Pistorio A, Besana C, Foti T, Ruperto N (2005). Preliminary diagnostic guidelines for macrophage activation syndrome complicating systemic juvenile idiopathic arthritis. J Pediatr.

[20] Tibaldi J, Pistorio A, Aldera E, Puzone L, El Miedany Y, Pal P (2020). Development and initial validation of a composite disease activity score for systemic juvenile idiopathic arthritis. Rheumatology (Oxford).

[21] Brachat AH, Grom AA, Wulffraat N, Brunner HI, Quartier P, Brik R (2017). Early changes in gene expression and inflammatory proteins in systemic juvenile idiopathic arthritis patients on canakinumab therapy. Arthritis Res Ther.

[22] Brunner HI, Quartier P, Alexeeva E, Constantin T, Koné-Paut I, Marzan K (2020). Efficacy and safety of canakinumab in patients with systemic juvenile idiopathic arthritis with and without fever at baseline: results from an open-label, active-treatment extension study. Arthritis Rheumatol.

[23] Iwata N, Nishimura K, Hara R, Imagawa T, Shimizu M, Tomiita M (2023). Long-term efficacy and safety of canakinumab in the treatment of systemic juvenile idiopathic arthritis in Japanese patients: results from an open-label phase III study. Mod Rheumatol.

[24] Pardeo M, Rossi MN, Pires Marafon D, Sacco E, Bracaglia C, Passarelli C (2021). Early treatment and IL1RN single-nucleotide polymorphisms affect response to anakinra in systemic juvenile idiopathic arthritis. Arthritis Rheumatol.

[25] Pardeo M, Bracaglia C, De Benedetti F (2017). Systemic juvenile idiopathic arthritis: new insights into pathogenesis and cytokine directed therapies. Best Pract Res Clin Rheumatol.

[26] Suematsu S, Matsuda T, Aozasa K, Akira S, Nakano N, Ohno S (1989). IgG1 plasmacytosis in interleukin 6 transgenic mice. Proc Natl Acad Sci U S A.

[27] De Benedetti F, Rucci N, Del Fattore A, Peruzzi B, Paro R, Longo M (2006). Impaired skeletal development in interleukin-6-transgenic mice: a model for the impact of chronic inflammation on the growing skeletal system. Arthritis Rheum.

[28] Fantuzzi G, Dinarello CA (1996). The inflammatory response in interleukin-1 beta-deficient mice: comparison with other cytokine-related knock-out mice. J Leukoc Biol.

[29] Fattori E, Cappelletti M, Costa P, Sellitto C, Cantoni L, Carelli M (1994). Defective inflammatory response in interleukin 6-deficient mice. J Exp Med.

[30] Shimamoto K, Ito T, Ozaki Y, Amuro H, Tanaka A, Nishizawa T (2013). Serum interleukin 6 before and after therapy with tocilizumab is a principal biomarker in patients with rheumatoid arthritis. J Rheumatol.

[31] Chakraborty A, Tannenbaum S, Rordorf C, Lowe PJ, Floch D, Gram H (2012). Pharmacokinetic and pharmacodynamic properties of canakinumab, a human anti-interleukin-1β monoclonal antibody. Clin Pharmacokinet.

[32] Kostik MM, Isupova EA, Belozerov K, Likhacheva TS, Suspitsin EN, Raupov R (2022). Standard and increased canakinumab dosing to quiet macrophage activation syndrome in children with systemic juvenile idiopathic arthritis. Front Pediatr.

[33] Lainka E, Baehr M, Raszka B, Haas JP, Hügle B, Fischer N (2021). Experiences with IL-1 blockade in systemic juvenile idiopathic arthritis - data from the German AID-registry. Pediatr Rheumatol Online J.

[34] Sota J, Vitale A, Insalaco A, Sfriso P, Lopalco G, Emmi G (2018). Safety profile of the interleukin-1 inhibitors anakinra and canakinumab in real-life clinical practice: a nationwide multicenter retrospective observational study. Clin Rheumatol.

